# Synthesis of clinical prediction models under different sets of covariates with one individual patient data

**DOI:** 10.1186/s12874-015-0087-x

**Published:** 2015-11-19

**Authors:** Daisuke Yoneoka, Masayuki Henmi, Norie Sawada, Manami Inoue

**Affiliations:** Department of Statistical Science, School of Multidisciplinary Sciences, SOKENDAI (The Graduate University for Advanced Studies), Tokyo, Japan; Department of Data Science, The Institute of Statistical Mathematics, Tokyo, Japan; Research Center for Cancer Prevention and Screening, National Cancer Center, Tokyo, Japan; Department of Global Health Policy, Graduate School of Medicine, The University of Tokyo, Tokyo, Japan

**Keywords:** Research synthesis, Clinical prediction model, Multivariate meta-analysis, Model misspecification

## Abstract

**Background:**

Recently, increased development of clinical prediction models has been reported in the medical literature. However, evidence synthesis methodologies for these prediction models have not been sufficiently studied, especially for practical situations such as a meta-analyses where only aggregated summaries of important predictors are available. Also, in general, the covariate sets involved in the prediction models are not common across studies. As in ordinary model misspecification problems, dropping relevant covariates would raise potentially serious biases to the prediction models, and consequently to the synthesized results.

**Methods:**

We developed synthesizing methods for logistic clinical prediction models with possibly different sets of covariates. In order to aggregate the regression coefficient estimates from different prediction models, we adopted a generalized least squares approach with non-linear terms (a sort of generalization of multivariate meta-analysis). Firstly, we evaluated omitted variable biases in this approach. Then, under an assumption of homogeneity of studies, we developed bias-corrected estimating procedures for regression coefficients of the synthesized prediction models.

**Results:**

Numerical evaluations with simulations showed that our approach resulted in smaller biases and more precise estimates compared with conventional methods, which use only studies with common covariates or which utilize a mean imputation method for omitted coefficients. These methods were also applied to a series of Japanese epidemiologic studies on the incidence of a stroke.

**Conclusions:**

Our proposed methods adequately correct the biases due to different sets of covariates between studies, and would provide precise estimates compared with the conventional approach. If the assumption of homogeneity within studies is plausible, this methodology would be useful for incorporating prior published information into the construction of new prediction models.

## Background

Development of accurate clinical prediction models is a relevant issue in medical research, and the number of published prediction models has increased substantially over the last few decades. For example, the literature already contains 102 proposed risk prediction models for cardiovascular disease [1] and 25 for the risk of developing type 2 diabetes (including 11 logistic regression models) [2]. However, many authors point out that these developed prediction models are not necessarily accurate and cannot be generalized well to larger, broad populations [3–5]. One of the most relevant reasons is imprecise estimates due to substantially insufficient samples compared with the number of involved predictors in the development of prediction models [3]. Therefore, the research synthesis methodologies of clinical prediction models have received interest in terms of achieving more accurate estimations by using larger datasets. Debray et al. [6] addressed the issue of synthesizing results by proposing a multivariate meta-analysis approach to combining regression coefficients of published logistic prediction models. The concept of using this approach for the synthesis of regression coefficients, which can be regarded as a variant of the generalized least squares method by Becker and Wu [7], explicitly takes into account the distinction of within- and between-study covariance of coefficients, and it should provide a valid solution when complete and unbiased estimates of the regression coefficients are available. However, in general, each published prediction model is developed using different sets of covariates [7–10], and dropping relevant covariates would raise potentially serious biases to the prediction models, as in ordinary model misspecification problems. To tackle this problem, the Fibrinogen Studies Collaboration [11] proposed a multivariate meta-analysis approach to borrow strength from partially adjusted results by using individual patient data (IPD), and Riley et al. [12] demonstrated the approach in practice. In addition, Resche-Rigon et al. [13] adopted a multiple imputation method with IPD. On the other hand, instead of using every IPD record, Debray et al. [6] considered a method that uses the reported summary statistics with one set of IPD. They adopted an ad-hoc approach utilizing mean or zero imputations for the missing coefficient estimates to straightforwardly apply the multivariate meta-analysis method [6]. Although Debray’s approach is a simple implementation strategy, it should raise substantial biases to the synthesized results because the interpretation of the coefficients depends on which covariates are included in each regression model.

In this article, we develop valid inference methods for synthesizing regression coefficients of published prediction models under different sets of covariates. We provide bias assessment methods for regression coefficients when important predictors are dropped in some studies, and thereby supply bias-corrected estimators for the synthesized prediction models under the assumption of the homogeneity of studies in meta-analysis. We show that our method is asymptotically more efficient than the conventional approach applying multivariate meta-analysis, by using studies with common covariate sets and the previously proposed approach of Debray et al. [6]. Further, we demonstrate the robustness property against the misspecification of within-study covariance matrices. While we discuss here the synthesis of logistic prediction models, our approach could be extended to more general cases such as survival prediction models [14].

The rest of the paper is organized as follows. In Section ‘Methods’, we consider as the first step the problem of omitted variable bias in the logistic regression model. Then we propose a non-linear model with the terms of omitted variable bias to synthesize the published coefficients, where the generalized nonlinear least squares (GNLS) method is applied for estimation. We also show that our method has desirable properties (i.e., efficiency and robustness). In Section ‘Results: simulation studies’, the performance of our method is numerically checked by simulation studies. Our method is illustrated through the use of a practical dataset in Section ‘Results: application in risk prediction models for occurrences of stroke’. This dataset was obtained from epidemiological studies on the incidence of stroke; these studies were conducted in Japan and contain several covariates. Each study separately analyzed with logistic regression models on each cohort, but the covariates in the models are unbalanced across cohorts. Finally, Section ‘Discussion’ provides some discussion and an examination of future problems.

## Methods

We consider a similar situation as Debray et al. [6] in that we can use reported summary statistics from previous logistic regression models with different sets of covariates and at least one IPD from the publications or the authors themselves. Suppose that each published prediction model has a subset of covariates in the IPD, and is constructed for same prediction task. The number of published prediction models is *N* ($i=1,\dots,N$) and the *i*th article reports the estimated coefficients, $\hat {\boldsymbol {\theta }}_{i}$, and the covariance matrix $\boldsymbol {\Sigma }_{i}=\text {Cov}(\hat {\boldsymbol {\theta }}_{i})$ (at least its diagonal elements). Each $\hat {\boldsymbol {\theta }}_{i}$ is a column vector of possibly different length, $\hat {\boldsymbol {\theta }}_{i}=(\hat {\theta }_{i1},\dots,\hat {\theta }_{i p_{i}})^{T}$, and *p*_*i*_ represents the differences of covariate sets among studies. To synthesize these regression coefficients, Debray et al. [6] utilize the mean or zero imputation methods for omitted coefficients and apply the technique of multivariate meta-analysis. Another simple approach is to apply multivariate meta-analysis using studies with common covariate sets [6]. However, the former approach leads to biased results and the latter is not biased but leads to loss of efficiency by ignoring indirect information from omitted studies. In order to improve this situation, we propose a new method for synthesis of logistic regression coefficients under different sets of covariates.

For simplicity, we assume the case where the true model has the full set of covariates in the IPD, which means the prior models have subsets of covariates and are considered as under-specified models. Note that since the omitted variables from the true models (full models) may be correlated with the included variables, the subset models are confounded and biased compared with true models. Our method can be generalized to more complex cases where the previous models for meta-analysis are a mixture of under- and over-specified models. Our method can be also applied to more general models such as generalized linear models, including linear regression and other non-linear regression models with some modification. This study was approved by the Institutional Review Board at the National Cancer Center in Tokyo and The University of Tokyo, Japan.

### The omitted variable bias in the logistic regression model

Firstly, we introduce the omitted variable bias under one original logistic regression setting, which can afterward be extended to the meta-analysis setting with the assumption that the covariate sets differ among studies.

Let $\boldsymbol {X}=\left (1,X_{2}, \dots, X_{j}\right)^{T}$ and $\boldsymbol {Z}=(Z_{1}, \dots, Z_{k})^{T}$ be vectors of covariates and *Y*∈{0,1} be a binary response variable. Suppose the data-generating process (DGP) can be formulated by the true model:

(1) $$\begin{array}{@{}rcl@{}} \text{logit} P(Y=1|\boldsymbol{X},\boldsymbol{Z})=\boldsymbol{X}^{T}\boldsymbol{\alpha}+\boldsymbol{Z}^{T}\boldsymbol{\beta}, \end{array} $$

where ***α***,***β*** are the true parameters of interest and “logit” means the logistic function, $\text {logit}(p)=\text {log} \left (p/(1-p)\right)$. The misspecified model is assumed to be fitted, which omits relevant covariates ***Z*** from the true model (1). Specifically,

(2) $$\begin{array}{@{}rcl@{}} \text{logit}\,\, P(Y=1|\boldsymbol{X})=\boldsymbol{X}^{T}\boldsymbol{\gamma}. \end{array} $$

We investigate the degree to which the regression coefficient ***γ***, estimated under the misspecified model, differs from the true parameters ***α***,***β***, and define the differences as the omitted variable bias.

To derive the omitted variable bias, the unbiasedness condition of the estimating function can be employed [15]; this approach is a generalized result of the landmark paper of White [16]. In the meta-analysis framework, the idea of the omitted variable bias can function as an analogy of different covariate sets and as a representation of the incorporation of indirect information from prior models.

In general, score functions from misspecified models cannot satisfy the unbiasedness condition of estimating functions. Therefore, the first step is to find the solution of the unbiasedness condition of estimating function (3), i.e., find ***γ***^∗^=***f***(***α***,***β***,*p*_***XZ***_), which is the function of the true parameters ***α***,***β*** and the joint distribution of covariates, *p*_***XZ***_;

(3) $$\begin{array}{@{}rcl@{}} E\left[\left\{Y-\frac{1}{1+\exp\left(-\boldsymbol{X}^{T}\boldsymbol{\gamma}^{*}\right)}\right\} \boldsymbol{X}\right] = 0. \end{array} $$

Here, the expectation is taken by the true joint distribution of *Y*, ***X*** and ***Z*** defined from (1) and *p*_***XZ***_. Under some regularity conditions, the maximum likelihood estimate of ***γ*** from the misspecified model (2) is a consistent estimate of ***γ***^∗^.

Secondly, for assessing biases caused by dropping the important predictors, we assume to have (at least) one IPD with the outcome and the full covariates ***X***, ***Z***. This assumption is considered reasonable for researchers who want to develop a new prediction model on their own IPD, incorporating prior summary statistics from regression results. Using the IPD, we can empirically solve (3) and derive the omitted variable bias.

Note that in the general case, the function ***f*** cannot be written in closed form due to its nonlinearity, but in the following case where every omitted covariate is a continuous variable it can be explicitly written.

### Special case : omitted covariates, ***Z***, are continuous variables

In general, the maximum likelihood estimate of ***γ*** in (2) (consistently) estimates ***γ***^∗^ as the solution of (3). In particular, for the cases of normal continuous variables, the following analytical evaluation can be adapted. Now we suppose ***Z***|***X*** follows the multivariate normal distribution, *N*(***μ***_***Z|X***_,***Ω***_***Z|X***_). Based on the normality assumption of ***Z|X***, we have

$$\begin{array}{@{}rcl@{}} \boldsymbol{Z}=\boldsymbol{\Delta}\boldsymbol{X}+\boldsymbol{\tau} \end{array} $$

where $\boldsymbol {\Delta }=(\boldsymbol {\delta }_{1}, \dots, \boldsymbol {\delta }_{k})^{T}$ is *k*×*j* matrix and ***τ***∼*N*_***τ***_(***0***,***Ω***_***Z|X***_).

Applying the technique of Chao et al. [17] to our covariate structure and using the probit approximation of logistic distribution, the expectation of *Y* conditional on ***X*** can be expressed as follow:

$${\fontsize{7.3}{6}\begin{aligned} E[\!Y|\boldsymbol{X}]=P(Y=1|\boldsymbol{X})&=\int \frac{1}{1+\exp\left(-\boldsymbol{X}^{T}\boldsymbol{\alpha}-(\boldsymbol{\Delta}\boldsymbol{X}+\boldsymbol{\tau}\right)^{T} \boldsymbol{\beta})}N_{\boldsymbol{\tau}}(0,\boldsymbol{\Omega}_{\boldsymbol{Z|X}}) d\boldsymbol{\tau}\\ &\approx \Phi \left[c \left\{ \frac{\boldsymbol{X}^{T}(\boldsymbol{\alpha}+\boldsymbol{\Delta}^{T}\boldsymbol{\beta})}{\sqrt{1+c^{2}\boldsymbol{\beta}^{T}\boldsymbol{\Omega}_{\boldsymbol{Z|X}}\boldsymbol{\beta}} }\right\}\right], \end{aligned}} $$

where *Φ* is the cumulative distribution function of standard normal distribution and *c*=16(3)^1/2^/15*π* is the adjustment factor for probit approximation of the logistic distribution proposed by Johnson et al. [18].

In order to satisfy the unbiasedness condition of the estimating function, (3), we have

$${ \fontsize{9.1}{6}\begin{aligned} E \left[\Phi \left[c \left\{ \frac{\boldsymbol{X}^{T}\left(\boldsymbol{\alpha}+\boldsymbol{\Delta}^{T}\boldsymbol{\beta}\right)}{\sqrt{1+c^{2}\boldsymbol{\beta}^{T}\boldsymbol{\Omega}_{\boldsymbol{Z|X}}\boldsymbol{\beta}} }\right\}\right] \boldsymbol{X}- \Phi \left\{c\left(\boldsymbol{X}^{T}\boldsymbol{\gamma}^{*}\right)\right\}\boldsymbol{X}\right]=0 \end{aligned}} $$

Therefore, the function ***f*** should be denoted as

(4) $$\begin{array}{@{}rcl@{}} \boldsymbol{\gamma^{*}}=\boldsymbol{f}(\boldsymbol{\alpha},\boldsymbol{\beta}, p_{\boldsymbol{XZ}}) \approx \frac{\boldsymbol{\alpha}+\boldsymbol{\Delta}^{T}\boldsymbol{\beta}}{\sqrt{1+c^{2}\boldsymbol{\beta}^{T}\boldsymbol{\Omega}_{\boldsymbol{Z|X}}\boldsymbol{\beta}}} \end{array} $$

which is the generalization of the results of Chao et al. [17] and Cramer et al. [19].

### Nonlinear model for meta-analysis

Suppose there exist *N* reported models ($i=1,\dots,N$) with their estimated coefficients of ***α***,***β*** and ***γ*** and their covariance matrices, and when $i=1,\dots,N$, studies fit the true model (1) with a full set of covariates, ***X*** and ***Z***, and when $i=n+1,\dots, N$, studies mistakenly omit covariates ***Z***. We assume the homogeneity of studies (i.e., the distribution of covariates and outcomes are common across the studies in the meta-analysis). Here we show only the case where ***Z*** is omitted, but the case where ***X*** is omitted can be considered in the same manner, and further, it is easy to generalize to various other omittance patterns. To synthesize the estimated coefficients vectors from the logistic regression models, we apply a GNLS method to incorporate the unequal variances of studies into meta-analysis.

Based on this setting, the nonlinear model for meta-analysis can be formulated as follows;

(5) $$\begin{array}{@{}rcl@{}} \hat{\boldsymbol{\theta}}_{i}=\boldsymbol{g}_{i}(\boldsymbol{\alpha}, \boldsymbol{\beta}, p_{\boldsymbol{XZ}})+\boldsymbol{\varepsilon}_{i} \ \ \ \ \ (i=1,\dots,n), \end{array} $$

where

$$\begin{array}{@{}rcl@{}} \boldsymbol{g}_{i}(\boldsymbol{\alpha}, \boldsymbol{\beta}, p_{\boldsymbol{XZ}}) =\left\{\begin{array}{ll} (\boldsymbol{\alpha}^{T},\boldsymbol{\beta}^{T})^{T} &(i=1,\dots,n) \\ \boldsymbol{f}(\boldsymbol{\alpha}, \boldsymbol{\beta}, p_{\boldsymbol{XZ}}) & (i=n+1,\dots,N), \end{array}\right., \\ \left(\begin{array}{c} \boldsymbol{\varepsilon}_{1} \\ \vdots \\ \boldsymbol{\varepsilon}_{N} \end{array} \right) \sim N(\boldsymbol{0},\boldsymbol{\Sigma}), \ \ \boldsymbol{\Sigma}=\left[\begin{array}{ccc} \text{Cov}(\hat{\boldsymbol{\theta}}_{1}) & \ldots & \boldsymbol{0}\\ \vdots & \ddots & \vdots\\ \boldsymbol{0} &\ldots & \text{Cov}(\hat{\boldsymbol{\theta}}_{N}) \end{array}\right], \end{array} $$

and $\hat {\boldsymbol {\theta }}_{i}$ is the column vector of reported coefficients in the *i*th study. The function ***f***() comes from the omitted variable bias formula introduced in the previous section, whose formulation is reasonable if an assumption of homogeneity of studies in meta-analysis is acceptable.

In a large sample, the estimated coefficients $\hat {\boldsymbol {\theta }}_{i}$ are (approximately) normally distributed with mean ***θ***_*i*_=***g***_*i*_(***α***,***β***,*p*_***XZ***_) and covariance $\text {Cov}(\hat {\boldsymbol {\theta }}_{i})$. This asymptotic normality of estimated coefficients leads to the justification of the GNLS approach.

Under the model (5), overall estimates of the regression coefficients $\hat {\boldsymbol {\alpha }}^{*}$ and $\hat {\boldsymbol {\beta }}^{*}$ can be obtained by GNLS as follows:

$$\begin{array}{@{}rcl@{}} \left(\hat{\boldsymbol{\alpha}}^{*T},\hat{\boldsymbol{\beta}}^{*T}\right)^{T} &=& \mathop{\arg\min}_{\boldsymbol{\alpha},\boldsymbol{\beta}} \sum_{i=1}^{N}\left\{\hat{\boldsymbol{\theta}}_{i} -\boldsymbol{g}_{i}(\boldsymbol{\alpha}, \boldsymbol{\beta}, \hat{p}_{\boldsymbol{XZ}})\right\}^{T}\\&&\times\boldsymbol{\Sigma}^{-1} \left\{\hat{\boldsymbol{\theta}}_{i} - \boldsymbol{g}_{i}(\boldsymbol{\alpha}, \boldsymbol{\beta}, \hat{p}_{\boldsymbol{XZ}})\right\}, \end{array} $$

where $\hat {p}_{\boldsymbol {XZ}}$ is an estimate of *p*_***XZ***_ from the IPD.

The diagonal of the covariance matrix ***Σ*** is typically reported in the literature but the off-diagonals are unknown, thus off-diagonal elements can be imputed by using the IPD. We employ the same imputation method as Debray et al. [6] based on the IPD as follows;

$$\begin{array}{@{}rcl@{}} \text{Cov} (\hat{\boldsymbol{\theta}}_{i,W})=\hat{V}_{i}^{\frac{1}{2}}R_{IPD}\hat{V}_{i}^{\frac{1}{2}}, \end{array} $$

where $\text {Cov} (\hat {\boldsymbol {\theta }}_{i,W})$ is a working covariance matrix of the *i*th study which is applied to one of the block diagonal elements of ***Σ***, $\hat {V}_{i}=\text {diag}(\text {Cov} (\hat {\boldsymbol {\theta }}_{i}))$ is a diagonal matrix whose diagonal elements are the estimated standard errors (SE) reported from each study and *R*_*IPD*_ is a working correlation matrix of coefficients calculated from the IPD. The covariance matrix can be calculated with a sandwich estimator under the model misspecification assumption instead of the imputation based on the IPD [15], but there computational complexity remains a problem and little improvement is gained in simulations studies. Furthermore, even if the covariance matrix is misspecified, the proposed estimator is still consistent and asymptotically normally distributed with a sandwich covariance matrix. This robustness follows the asymptotic theory of the generalized estimating equations. In this situation, let $\hat {\boldsymbol {\alpha }}_{W}$ and $\hat {\boldsymbol {\beta }}_{W}$ denote our estimators with the working covariance matrix. The covariance matrix of these estimators can be estimated by

$$\begin{array}{@{}rcl@{}} \left(\hat{\boldsymbol{D}}^{T}\boldsymbol{\Sigma}_{W}^{-1}\hat{\boldsymbol{D}}\right)^{-1} \hat{\boldsymbol{D}}^{T} \boldsymbol{\Sigma}_{W}^{-1} \text{Cov}\left(\hat{\boldsymbol{\theta}}_{I}\right) \boldsymbol{\Sigma}_{W}^{-1} \hat{\boldsymbol{D}} \left(\hat{\boldsymbol{D}}^{T} \boldsymbol{\Sigma}_{W}^{-1} \hat{\boldsymbol{D}}\right)^{-1}, \end{array} $$

where $\hat {\boldsymbol {D}}=\left (\hat {\boldsymbol {D}}_{1}^{T},\dots,\hat {\boldsymbol {D}}_{N}^{T}\right)^{T}, \hat {\boldsymbol {D}}_{i}=\partial \boldsymbol {g}_{i}\left (\boldsymbol {\alpha }, \boldsymbol {\beta }, \hat {p}_{\boldsymbol {XZ}}\right)/\partial \left (\boldsymbol {\alpha }^{T}, \boldsymbol {\beta }^{T}\right)|_{\left (\boldsymbol {\alpha }^{T}, \boldsymbol {\beta }^{T}\right)=\left (\hat {\boldsymbol {\alpha }}^{T}_{W}, \hat {\boldsymbol {\beta }}^{T}_{W}\right)}$, ***Σ***_*W*_ is a working covariance matrix, and $\text {Cov}(\hat {\boldsymbol {\theta }}_{I})=\left (\left \{\hat {\boldsymbol {\theta }}_{i} - \boldsymbol {g}_{i}\left (\hat {\boldsymbol {\alpha }}_{W}, \hat {\boldsymbol {\beta }}_{W}, \hat {p}_{\boldsymbol {XZ}}\right)\right \}\left \{\hat {\boldsymbol {\theta }}_{i} - \boldsymbol {g}_{i}\left (\hat {\boldsymbol {\alpha }}_{W}, \hat {\boldsymbol {\beta }}_{W}, \hat {p}_{\boldsymbol {XZ}}\right)^{T}\right \}\right)$ [20, 21]. This idea essentially comes from Liu et al. [21] and can be regarded as analogy of the result proposed by Chen et al. [22].

In addition to the above, if the working covariance matrix is a good approximation of the true covariance structure, the following relationship holds:

$$\begin{array}{@{}rcl@{}} \text{Avar} \left(\hat{\boldsymbol{\theta}}^{*}\right)\le \text{Avar} \left(\hat{\boldsymbol{\theta}}_{M}\right) \le \text{Avar} \left(\hat{\boldsymbol{\theta}}_{S}\right), \end{array} $$

where Avar denotes an asymptotic covariance matrix, and $\hat {\boldsymbol {\theta }}^{*}$, $\hat {\boldsymbol {\theta }}_{M}$ and $\hat {\boldsymbol {\theta }}_{S}$ are the estimates of $\boldsymbol {\theta }=\left (\boldsymbol {\alpha }^{T},\boldsymbol {\beta }^{T}\right)^{T}$ obtained from our proposed method, from multivariate meta-analysis using only the studies with full covariates and from a single study with full covariates, respectively, when the number of studies *N* goes to infinity.

Here, we assume a fixed effect model which presumes that there is no heterogeneity in the distribution of covariates and in the values of the parameters of interest. This assumption may sometimes be unrealistic. Therefore, we recommend considering whether this assumption is reasonable based on background knowledge or reported information. In addition, we can propose how to modify this to a random effects model to incorporate the heterogeneity by assuming that the parameters underlying studies and the parameters of distribution of covariates follow some distribution. For example, considering the case that all omitted variables are continuous (i.e., Section ‘The omitted variable bias in the logistic regression model)’, we can incorporate random effects by assuming that ***α***,***β***, ***Δ*** and ***Ω***_***Z|X***_ in (4) follow distributions. Random effects in ***α***,***β*** accommodate the heterogeneity of parameters and random effects in ***Δ*** and ***Ω***_***Z|X***_ accommodates that of distribution of covariates.

## Results: simulation studies

### Simulation setup

In this section we describe a Monte Carlo simulation which was performed to evaluate the performance of our proposed method. In the simulation, we empirically calculate the omitted variable bias by using Eq. (3) instead of Eq. (4). The parameters which varied in the simulation scenario were the true value of a parameter in a DGP model, the number of predictors and the distribution of covariates (continuous/discrete covariates). For simplicity, we examined the case where the number of predictors in the models in this simulation was 1 or 2 (i.e. *X*_1_, *X*_2_ or both). The DGP model was logit*P*(*Y*=1|*X*_1_,*X*_2_)=*α*_0_+*α*_1_*X*_1_+*α*_2_*X*_2_. For checking the sensitivity for the true value of the parameter in the DGP model, *α*_1_ varied from -2 to 1, and true values of other parameters was set at 1 (i.e. *α*_0_=*α*_2_=1).

We simulated $N=9, \ (i=1,\dots,9)$ independent studies with 100 samples in each, and of these studies, 3 studies (*i*=1,2,3) included a full set of covariates (*X*_1_ and *X*_2_), 3 (*i*=4,5,6) were supposed to omit *X*_1_ and 3 (*i*=7,8,9) were supposed to omit *X*_2_. One of the studies with the full set of covariates was used as the IPD. As mentioned above, the off-diagonals of the covariance matrix were often unknown, thus we adopted the imputation by IPD proposed in the Methods section. In this simulation, we compared the performance of this imputation with the setting using a true covariance structure, which could be estimated from simulation settings.

We classified the scenario into 2 cases according to the distribution of covariates (continuous/discrete distribution). In Case 1, *X*_1_ and *X*_2_ were both continuous and followed the multivariate normal distribution, $X_{1},X_{2} \sim N\left (\left (\begin {array}{l} 2 \\ 2\end {array}\right), \left (\begin {array}{ll} 1 & r \\ r &1 \end {array}\right)\right)$. The correlation, *r*, between *X*_1_ and *X*_2_ was set at 0 or 0.5. In this case, we checked the performance of the approximation formula (4). Case 2 was the more practical case in which continuous and discrete distributions were mixed (i.e., *X*_1_ was continuous and *X*_2_ was binary). *X*_2_ was binarized from the distribution in Case 1 by a threshold value set at 2. Under these settings, 1000 Monte Carlo simulations were implemented. If the models could not be fitted and converged, their results were excluded from the calculation of bias and mean squared error (MSE).

Performance of the proposed method was evaluated by bias and MSE, comparing it with two ordinary methods. M1 was the multivariate meta-analysis using only 3 studies with a full set of covariates. From a theoretical perspective, the M1 strategy does not include any bias but is inferior in efficiency compared with our proposed method, which can be checked using the results of MSE. M2 was the multivariate meta-analysis after mean imputation of missing coefficients, whose method was proposed in Debray et al. [6]. For example, coefficients and their estimated standard errors of *X*_2_ from 3 studies (*i*=4,5,6) were imputed by the means of the other 6 studies. We tried the zero imputation method, which Debray et al. adopted [6] and called uninformative regression coefficients, but it did not show notable results compared with the results from M2 (mean imputation). Therefore, we decided not to include the results of this method.

### Simulation results

The results of the simulation revealed that compared with the ordinary meta-analysis, our proposed estimator generally produced more precise and less-biased estimates for all simulation settings (Table 1). The rationale of small proportion of convergence at *α*_1_=1 was that under the simulation setting, *P*(*Y*=1) could be over 0.9, which led to small number of event at a single study. The bias of our estimator ranged from -0.052 to 0.097 (mean: 0.021) for Case 1 and from -0.064 to 0.488 (mean: 0.040) for Case 2. The MSE of our estimator ranged from 0.021 to 0.803 (mean: 0.124) for Case 1 and from 0.012 to 0.486 (mean: 0.091) for Case 2. Although the M2 strategy in Case 1 and *r*=0 yielded somewhat biased results, the greatest amount of variation seemed to arise from the biased estimates of *α*_0_ in the models from which *X*_2_ was omitted.

**Table 1:** Performance of our proposed method on simulation data (Bias and MSE)

	Case 1; Both *X*_1_ and *X*_2_ are continuous											Case 2; *X*_1_ is continuous and *X*_2_ is binary								
		Correlation r=0					Correlation r=0.5					Correlation r=0					Correlation r=0.5			
Covariance matrix imputed with IPD																				
		-2	-1	0	1		-2	-1	0	1		-2	-1	0	1		-2	-1	0	1
Bias	Proposed method																			
	*α*_0_	-0.002	0.024	0.030	0.000		0.028	0.029	0.043	0.052		0.025	0.022	0.013	-0.040		0.057	0.037	0.006	-0.006
	*α*_1_	-0.052	-0.022	-0.001	0.086		-0.049	-0.024	-0.011	0.083		-0.050	-0.015	-0.003	0.071		-0.064	-0.017	0.002	0.025
	*α*_2_	0.038	0.022	0.012	0.097		0.030	0.018	0.010	0.058		0.044	0.007	0.018	0.266		0.049	0.001	0.012	0.488
	M1: Full set only																			
	*α*_0_	0.011	0.010	-0.018	-0.365		0.009	-0.009	-0.029	-0.058		0.011	0.016	-0.004	-0.016		0.027	0.011	-0.007	-0.002
	*α*_1_	-0.009	0.003	0.011	0.061		0.001	0.002	0.000	0.082		-0.001	0.002	0.000	0.029		-0.006	0.001	-0.004	0.012
	*α*_2_	0.001	-0.009	-0.019	0.061		0.001	0.000	-0.015	0.023		0.008	-0.027	-0.006	0.124		0.027	-0.008	0.001	0.272
	M2: Mean imputation																			
	*α*_0_	2.166	1.551	-0.193	-1.275		1.197	0.724	-0.145	-0.590		2.930	2.116	0.048	-1.131		2.612	1.789	0.050	-0.841
	*α*_1_	-1.807	-0.818	-0.171	0.218		-1.921	-1.101	-0.415	-0.135		-1.334	-0.756	-0.082	0.203		-1.385	-0.800	-0.142	0.075
	*α*_2_	0.811	0.218	-0.034	0.226		1.543	0.940	0.109	-0.145		0.034	-0.138	-0.069	-0.460		1.292	0.778	0.010	-1.356
MSE	Proposed method																			
	*α*_0_	0.123	0.098	0.205	0.803		0.085	0.056	0.101	0.251		0.096	0.055	0.065	0.136		0.074	0.044	0.047	0.086
	*α*_1_	0.045	0.021	0.037	0.215		0.060	0.025	0.057	0.181		0.051	0.015	0.012	0.058		0.057	0.017	0.016	0.059
	*α*_2_	0.033	0.022	0.034	0.204		0.044	0.027	0.060	0.188		0.144	0.055	0.051	0.247		0.173	0.070	0.076	0.486
	M1: Full set only																			
	*α*_0_	0.248	0.208	0.453	7.960		0.160	0.131	0.274	0.707		0.195	0.116	0.142	0.300		0.169	0.099	0.108	0.197
	*α*_1_	0.068	0.033	0.068	0.620		0.075	0.033	0.094	0.398		0.096	0.027	0.025	0.112		0.099	0.030	0.028	0.112
	*α*_2_	0.038	0.032	0.093	0.591		0.045	0.034	0.118	0.570		0.162	0.077	0.103	2.165		0.190	0.095	0.120	4.883
	M2: Mean imputation																			
	*α*_0_	5.290	2.663	0.236	1.929		1.633	0.625	0.113	0.638		8.771	4.532	0.039	1.372		6.912	3.242	0.033	0.775
	*α*_1_	3.694	0.806	0.071	0.242		3.771	1.254	0.214	0.213		2.083	0.601	0.012	0.126		2.075	0.661	0.026	0.087
	*α*_2_	0.870	0.195	0.088	0.209		2.424	0.934	0.091	0.333		0.310	0.098	0.079	2.058		1.919	0.676	0.065	5.396
Convergence proportion (%)																				
		100	100	99.1	61.5		100	100	98.4	67.3		100	100	98.4	61.2		100	100	98.4	60.3
True Covariance matrix																				
		-2	-1	0	1		-2	-1	0	1		-2	-1	0	1		-2	-1	0	1
Bias	Proposed method																			
	*α*_0_	0.032	0.034	0.049	0.215		0.058	0.038	0.050	0.185		0.059	0.045	0.028	0.032		0.075	0.054	0.014	0.041
	*α*_1_	-0.118	-0.048	-0.005	0.099		-0.110	-0.046	-0.010	0.105		-0.116	-0.037	-0.004	0.072		-0.117	-0.036	0.003	0.036
	*α*_2_	0.070	0.055	0.052	0.104		0.055	0.049	0.058	0.096		0.082	0.036	0.039	0.250		0.072	0.023	0.037	0.462
MSE	Proposed method																			
	*α*_0_	0.111	0.089	0.196	0.690		0.077	0.053	0.094	0.204		0.091	0.054	0.065	0.123		0.071	0.043	0.046	0.080
	*α*_1_	0.050	0.021	0.034	0.161		0.061	0.024	0.050	0.125		0.057	0.015	0.012	0.050		0.062	0.017	0.015	0.051
	*α*_2_	0.031	0.022	0.032	0.146		0.039	0.026	0.053	0.136		0.133	0.049	0.049	0.215		0.145	0.060	0.069	0.417
Convergence proportion (%)																				
		100	100	99.1	61.5		100	100	98.4	67.3		100	100	98.4	61.2		100	100	98.4	60.3

The relative efficiency (RE) of the estimates of M1 versus those of our proposed method ranged from 1.023 to 9.913 (mean: 2.323) for Case 1 and from 1.098 to 10.047 (mean: 2.495) for Case 2. The RE of the estimates of M2 ranged from 1.025 to 82.069 (mean: 20.043) for Case 1 and from 0.600 to 93.405 (mean: 123.760) for Case 2.

In terms of the RE of the estimates from the true covariance structure versus the imputation method for unknown elements in the covariance structure, the RE of the covariance structure imputed from the IPD versus the true covariance structure ranged from 0.900 to 1.448 (mean: 1.126) for Case 1 and from 0.895 to 1.193 (mean: 1.065) for Case 2.

Comparing the MSE by correlation value (*r*=0 versus 0.5), in Case 1 the mean MSE of our proposed method was *r*=0: 0.074 versus *r*=0.5: 0.005. In Case 2, the mean MSE of our proposed method was *r*=0: 0.113 versus *r*=0.5: 0.174.

## Results: application in risk prediction models for occurrences of stroke

We applied the proposed methods to a series of epidemiologic studies that developed risk prediction models for occurrences of stroke. Stroke is one of the leading causes of death or physical/cognitive impairment in both developed and developing countries, and therefore numerous prediction models have been developed and many clinical characteristics have been identified as potential predictors [23, 24]. However, the overall influence of various risk factors is still unclear, with conflicting results from several reports [24].

### Application setup

We obtained 10 sets of IPD from studies conducted by the Japan Public Health Center-based Prospective Study (JPHC study). The JPHC study covers 11 public health center areas (Areas 1 – 11) across Japan. The total number of participants was 140,420, and the study population consisted of residents who were 40 to 69 years old at the time of the baseline survey. Details of the study design are well-documented in a previous report [25]. The outcome was confirmed according to the criteria provided by the National Survey of Stroke, which required a constellation of neurological deficits of sudden or rapid onset lasting at least 24 hours or until death [26, 27].

We fitted a logistic regression model to each available set of IPD and explored the important covariates related to patient characteristics and metabolic syndrome [28–30] such as age (years), time since last meal (minutes), body mass index (BMI (kg/ *m*^2^)), total cholesterol level (mg/dl), blood pressure (mmHg), cigarettes (per day), diabetes (yes/no), blood glucose (mg/dl), high-density lipoprotein (HDL (mg/dl)), and serum triglycerides (mg/dl) (Table 2). The sets of available covariates differed by region. For example, IPD from the Area 1 cohort did not include data on blood glucose, HDL or serum triglycerides since subjects in that cohort did not undergo any blood tests. One of our motivations in this study was to overcome this discrepancy among cohorts, which is typical in large-scale cohort studies investigating several outcomes.

**Table 2:** Estimated regression coefficients (and standard error) from JPHC data

	Area 1	Area 2	Area 3	Area 4	Area 5	Area 6
Sample	2121	1678	3396	859	3135	538
Incedence of stroke	109	82	132	23	142	35
Intercept	-12.280 (1.394)	-9.828 (1.668)	-9.917 (1.225)	-8.703 (2.846)	-11.940 (1.367)	-9.475 (3.070)
Age	0.085 (0.022)	0.095 (0.024)	0.066 (0.018)	0.022 (0.042)	0.113 (0.016)	0.126 (0.042)
Postprandial time	-0.016 (0.026)	-0.023 (0.041)	-0.019 (0.018)			
BMI	0.001 (0.001)	0.000 (0.001)	0.000 (0.001)	0.000 (0.002)	0.000 (0.001)	-0.002 (0.002)
Total cholesterol level	0.003 (0.003)	-0.001 (0.003)	-0.001 (0.003)	-0.001 (0.006)	-0.004 (0.003)	-0.011 (0.006)
Blood pressure	0.027 (0.005)	0.016 (0.007)	0.028 (0.005)	0.022 (0.012)	0.020 (0.006)	0.013 (0.010)
Smoke (per day)	0.020 (0.009)	0.028 (0.010)	0.010 (0.007)	0.012 (0.019)	0.000 (0.007)	-0.019 (0.021)
Diabetes	0.397 (0.504)	1.202 (0.525)	0.738 (0.362)	0.240 (1.314)	0.302 (0.314)	-0.174 (1.065)
Glucose			-0.004 (0.004)	0.012 (0.006)	0.004 (0.003)	
HDL					-0.005 (0.007)	0.012 (0.014)
Triglycerides			0.001 (0.001)		0.000 (0.001)	0.001 (0.003)
AUC	67.01	68.74	67.97	65.52	69.16	68.19
Brier score	7.71	7.72	7.65	8.07	7.78	7.68
Hosmer-Lemeshow	10.91	15.78*	14.01	101.45*	54.96*	17.70*
	Area 7	Area 8	Area 9	Area 10	Proposed	Conventional
Sample	1601	1731	1586	2725		
Incedence of stroke	85	90	90	52		
Intercept	-9.223 (1.710)	-8.413 (1.499)	-10.300 (1.729)	-10.500 (1.878)	-10.170 (0.633)	-9.408nn
Age	0.088 (0.020)	0.072 (0.018)	0.096 (0.021)	0.069 (0.020)	0.067nn	0.060n
Postprandial time	-0.009 (0.024)	-0.007 (0.025)	-0.006 (0.019)	0.018 (0.034)	0.013 (0.011)	0.017 (0.013)
BMI	-0.001 (0.001)	-0.002 (0.001)	-0.001 (0.001)	0.000nn	0.000nnn	0.000 (0.001)
Total cholesterol level	0.001 (0.004)	0.001 (0.003)	0.001 (0.004)	-0.003 (0.005)	-0.001 (0.001)	0.001 (0.002)
Blood pressure	0.011 (0.006)	0.015 (0.006)	0.015 (0.007)	0.017 (0.007)	0.017 (0.002)	0.011 (0.004)
Smoke (per day)	0.025 (0.010)	0.007 (0.010)	0.020 (0.009)	0.011 (0.010)	0.013 (0.004)	0.020 (0.006)
Diabetes	0.168 (0.455)	0.052 (0.485)	0.268 (0.490)	0.694 (0.465)	0.158 (0.180)	0.084 (0.262)
Glucose	0.009 (0.003)		0.004 (0.004)	-0.001 (0.008)	0.010 (0.001)	0.014 (0.002)
HDL	-0.022 (0.010)	-0.001 (0.009)	-0.013 (0.010)	0.005 (0.012)	-0.004 (0.005)	-0.008 (0.006)
Triglycerides	-0.003 (0.002)	-0.002 (0.002)	-0.001 (0.001)	0.002 (0.002)	0.000 (0.001)	0.000 (0.001)
AUC	68.32	69.47	68.29	67.28	68.01	67.24
Brier score	7.77	7.63	7.64	8.03	7.72	7.80
Hosmer-Lemeshow	58.60*	28.70*	25.38*	186.76*	21.13*	21.17*

Proposed: our proposed method; Conventional: conventional meta-analysis using only studies with a full set of covariates

Area 9 is IPD

^*^ *p*-value of Hosmer-Lemeshow test is less than 0.05

Coefficients from each model were stored as aggregated statistics, which could be regarded as prior studies for meta-analysis. In terms of handling sporadically missing data (average missing rate was 2.8 % with a standard deviation of 2.5 %), complete case analysis was executed. One cohort (Area 9) remained as IPD and one cohort (Area 11) was used as test data for prediction. Next, we compared our methodology with conventional multivariate meta-analysis using only studies with a full set of covariates and with results based solely on IPD data.

Lastly, new prediction models were constructed by plugging the synthesized coefficients into the models and checking the performance of each model using the test data.

The discriminant performance of the prediction models was measured by the area under the receiver operator characteristic curve (AUC) and the Brier score (BS) (multiplied by 100), both of which are indicators of the accuracy of the prediction model. A higher AUC indicates higher prediction accuracy, while the BS has an inverse relationship [31]. In addition, the model’s calibration was examined by the Hosmer-Lemeshow chi-squared statistic [32].

### Application results

The results demonstrated that our approach provided considerably narrower confidence intervals and slightly better prediction performance compared with conventional multivariate meta-analysis (Table 2). Our estimator reduced the SE by 38–53 and 56–71 % compared with the SE from conventional meta-analysis and from the IPD, respectively.

In terms of prediction performance, the prediction model constructed from the synthesized coefficients showed slight improvements over the conventional approach, particularly in BS. The AUC and BS were respectively increased by 1.1 and −1.0 *%* on average compared with conventional meta-analysis, and decreased by −0.4 and 1.0 *%* on average compared with the IPD. The improvements in prediction performance were relatively small because the cohort of test data was remarkably similar to other cohorts across Japan that were aggregated into summary statistics as previously published studies.

## Discussion

Along with increasing attention to prediction models, there has been higher demand for approaches to the meta-analysis of regression coefficients. However these methodologies are not well developed due to the many difficulties caused by the different settings used by various studies, and further research is still needed, particularly compared with conventional meta-analysis methods such as synthesizing mean differences, correlation and so on [7]. This study demonstrated a method to conduct the meta-analysis of regression coefficients with different covariate sets under the assumption of homogeneity of studies (i.e., it is applicable in cases where studies in the meta-analysis have similar distributions of covariates and outcomes). Although this study temporarily assumed the models with a full set of covariates as a true model, our approach can be generalized to any formulation of previous models even if they are over-/under-specified compared to a constructing model. We notice, however, that we need careful arguments about what is an appropriate covariate set. Further, the assumption that (at least) one IPD is available can be considered reasonable in the frequent case in which a single researcher wants to construct a new prediction model on his or her own IPD, incorporating prior regression results (but with such prior results reported just in the form of summary statistics). The minimal use of IPD (use of one IPD and other summary statistics) distinguishes our approach from that of the Fibrinogen Studies Collaboration [11]. They assume that both full and partial models are applied in each cohort by using its cohort IPD, and thus the estimation of the correlation of coefficients between full and partial models is applicable. As the future work, we need to study the relationship between our method and their one, and examine whether their method can be applicable to the situation we are considering. Regarding these discussions, our study can provide the following guidelines for practitioners about how to analyze prior models with their own IPD by recognizing the issue of omitted variable bias as the differences of sets of predictors between their constructing models and prior models: 1) the first step is to construct a new and temporal model on their own data set, and 2) the second step is to apply our method to synthesize the previous regression coefficients with their temporal model and then update the model and obtain more accurate estimators.

Our method proved robust against the misspecification of the covariance structure. Because of this property we can arbitrarily set the covariance matrix of coefficients and thus it is possible to avoid the argument, often discussed with methods such as that of Becker and Wu [7], on whether the full covariance matrix of coefficients should be reported or not. This robustness property can be considered as an analogical result provided by Liu et al. [21]. They provide a framework of meta-analysis under heterogeneity by using a confidence density function and reparametrization of the problem setting. Their approach utilizes the reparameterization connecting each study-specific parameter to the common parameter using the transformation function ***M***_*i*_, which is used as the omitted variable bias formula in our setting. However, they assume that the omitted covariates are fixed values and thus they can estimate ***M***_*i*_ without a consideration of the distribution of covariates. In contrast, our approach provides more general guidelines for treating missing covariates in the meta-analysis.

The simulation performed in this study illustrated that our method is unbiased and has greater efficiency than a conventional meta-analysis approach as well as the technique proposed by Debray et al. [6]. Although our estimator was most efficient if the covariance structure was truly specified, it maintained its efficiency even if we misspecified the covariance structure, with a loss of efficiency by misspecification of only around 10 %.

Finally, we demonstrated the practical use of our approach with medical data on stroke prediction. Although the improvement of accuracy of the prediction model was relatively small, the confidence intervals of synthesized coefficients were dramatically decreased because information from other studies helped improve efficiency. In the context of multivariate meta-analysis, it is well known that we can gain precision by borrowing strength from other partially reported results [33–35]. This implies that our methodology can be applied not only to prediction models but also to observational studies such as a case-control/cross-sectional study whose main purpose is to identify causal effects.

As a limitation of this study, our method was examined in only one practical dataset. Although this data includes over 100,000 samples, the population was Japanese only, and can thus be regarded as one group with small heterogeneity. This situation may not be representative of an ordinary meta-analysis because the majority of recent meta-analyses include several groups with large heterogeneity due to studies undertaken globally. We think, however, that we took this heterogeneity into account by incorporating random effects, as mentioned in the Methods section. We welcome the re-evaluation of our method in other practical cases. Another potential limitation is that we implicitly assumed that the distributions of covariates are the same between studies. The assumption is required to calculate the expectation in Eq. (3) for each study to derive the omitted variable bias formula, ***γ***^∗^=***f***(***α***,***β***,*p*_***XZ***_). The assumption of homogeneity can also be relaxed by incorporating random effects into parameters related to the distribution, as discussed in the Methods section. However, a random effect model obscures the objective of a meta-analysis because under this model, a global “average”İ effect and the effect prevailing in particular circumstances are not identical [36]. We need further research about how to incorporate random effects and its interpretation.

## Conclusions

This study proposed a correction method for the omitted variable bias due to different sets of covariates between literature models in the meta-analysis of regression coefficients. Our approach attained efficiency that was comparable to that of conventional approaches. This study should be useful for practitioners who want to develop their prediction model on their own dataset and incorporate prior regression results.

## Abbreviations

IPD: individual patient data
GNLS: generalized nonlinear least squares
DGP: data-generating process
MSE: mean squared error
JPHD: Japan Public Health Center-based prospective study
AUC: area under the receiver operator characteristic curve
BS: brier score
